# ASO Author Reflections: Illuminating Pancreatic Neuroendocrine Tumor Biology Through Real-Time Murine Modeling

**DOI:** 10.1245/s10434-026-19239-3

**Published:** 2026-02-10

**Authors:** Tao Gao, Young Ki Hong

**Affiliations:** 1 Department of Surgery, Cooper University Health Care, Camden, NJ USA; 2 Department of Surgery, Rowan University School of Medicine, Camden, NJ USA; 3 Camden Cancer Research Center, Camden, NJ USA; 4 MD Anderson Cancer Center at Cooper, Camden, NJ USA

## Past

Pancreatic neuroendocrine tumors (PNETs) represent a rare and biologically heterogeneous malignancy for which therapeutic options other than surgery remain limited, especially in advanced stages. Although substantial progress has been made in defining the molecular landscape of PNETs in recent years, translation of these insights into effective systemic therapies has remained lagged behind. A major barrier has been the lack of preclinical models that faithfully recapitulate tumor biology while permitting dynamic, noninvasive monitoring of tumor growth and dissemination.^1^

Traditional xenograft approaches in PNET research, including subcutaneous and heterotopic implantation models, are convenient but often fail to capture the tissue-specific microenvironment that influences tumor differentiation, growth kinetics, and immune interactions. Conversely, orthotopic pancreatic implantation more closely mirrors human disease but has historically been difficult to monitor longitudinally without invasive procedures or serial sacrifice of the experimental animals.^2^ These limitations have constrained mechanistic studies of PNET progression and impeded efficient preclinical evaluation of emerging therapeutic strategies.

To address these challenges, our study sought to fill a critical gap in the field by developing a biologically relevant and cost-effective murine PNET model that enables real-time visualization and systematic side-by-side comparison of tumor behavior across distinct implantation sites.^3^

## Present

In this study, we engineered a novel murine enteroendocrine tumor-derived STC-1 cell line that stably co-expresses firefly luciferase and enhanced green fluorescent protein. This dual-reporter system enables both whole-animal bioluminescent imaging and cellular-level fluorescence-based analyses. By comparing subcutaneous, renal capsule, and orthotopic pancreatic implantation routes, we systematically evaluated tumor growth dynamics, histopathologic fidelity, and imaging performance across commonly used xenograft platforms.

Our results demonstrate that orthotopic pancreatic implantation yields consistent tumor take rates, reproducible growth patterns, and histologic features that closely resemble well-differentiated human PNETs. Importantly, bioluminescent imaging provided sensitive, noninvasive, and longitudinal assessment of tumor burden, enabling direct comparison of growth kinetics across implantation sites within the same experimental framework. The ability to visualize tumor progression in real time represents a substantial advance over conventional endpoint-based approaches.

Furthermore, by integrating lineage tracing, live imaging, and histopathologic validation, this model bridges a critical translational gap between *in vitro* systems and genetically engineered mouse models.^4^ The approach offers a practical and scalable platform for studying tumor–microenvironment interactions, treatment response, and metastatic behavior in PNETs—areas where existing models in PNET research field have been particularly limited.

## Future

The development of a bioluminescent, orthotopic murine PNET model in this study creates multiple opportunities for future investigation. Immediate next steps include adapting this platform for application in immunocompetent and syngeneic settings to directly interrogate tumor–immune interactions and evaluate immunomodulatory therapies, an area of increasing clinical interest in neuroendocrine tumors.

While the bioluminescent IVIS imaging used in the current study enables noninvasive, real-time, and longitudinal monitoring of PNET growth across multiple animals simultaneously, incorporation of endoscopic ultrasound and high-resolution MRI in future studies may provide complementary approaches to improve anatomical resolution and volumetric accuracy, yielding a more comprehensive assessment of tumor characteristics.

Beyond its potential therapeutic applications, this model enables mechanistic studies of tumor evolution, niche-specific differentiation, and early metastatic dissemination. Integration with single-cell and spatial transcriptomic approaches could provide unprecedented insight into cellular heterogeneity and lineage plasticity during PNET progression. Ultimately, by improving the fidelity and accessibility of preclinical modeling, this work aims to accelerate translation of biologically informed therapies and improve outcomes for patients with this challenging disease.
